# Boosting with adjuvanted SCB-2019 elicits superior Fcγ-receptor engagement driven by IgG3 to SARS-CoV-2 spike

**DOI:** 10.1038/s41541-023-00791-y

**Published:** 2024-01-05

**Authors:** Wonyeong Jung, Dansu Yuan, Benjamin Kellman, Isabela Garrido da Silva Gonzalez, Ralf Clemens, Eveline Pipolo Milan, Eduardo Sprinz, José Cerbino Neto, Igor Smolenov, Galit Alter, Ryan P. McNamara, Sue Ann Costa Clemens

**Affiliations:** 1 Ragon Institute of MGH, MIT, and Harvard, Cambridge, MA USA; 2 Institute for Global Health, University of Siena, Siena, Italy; 3 International Vaccine Institute, Seoul, Republic of Korea; 4 Centro de Estudos e Pesquisa em Moléstias Infecciosas Ltda. (CEPCLIN), Natal, Brazil; 5 Hospital de Clínicas de Porto Alegre, Porto Alegre, Brazil; 6 D’Or Institute for Research and Education (IDOR), Rio de Janeiro, Brazil; 7 Clover Biopharmaceuticals, Chengdu, China; 8 Oxford Vaccine Group, Department of Pediatrics, University of Oxford, and the NIHR Oxford Biomedical Research Centre, Oxford, UK; 9 Siena University, Siena, Italy

**Keywords:** Translational research, Vaccines, Virology

## Abstract

With the continued emergence of variants of concern, the global threat of COVID-19 persists, particularly in low- and middle-income countries with limited vaccine access. Protein-based vaccines, such as SCB-2019, can be produced on a large scale at a low cost while antigen design and adjuvant use can modulate efficacy and safety. While effective humoral immunity against SARS-CoV-2 variants has been shown to depend on both neutralization and Fc-mediated immunity, data on the effectiveness of protein-based vaccines with enhanced Fc-mediated immunity is limited. Here, we assess the humoral profile, including antibody isotypes, subclasses, and Fc receptor binding generated by a boosting with a recombinant trimer-tag protein vaccine SCB-2019. Individuals who were primed with 2 doses of the ChAdOx1 vaccine were equally divided into 4 groups and boosted with following formulations: Group 1: 9 μg SCB-2019 and Alhydrogel; Group 2: 9 μg SCB-2019, CpG 1018, and Alhydrogel; Group 3: 30 μg SCB-2019, CpG 1018, and Alhydrogel; Group 4: ChAdOx1. Group 3 showed enhanced antibody FcγR binding against wild-type and variants compared to Groups 1 and 2, showing a dose-dependent enhancement of immunity conferred by the SCB-2019 vaccine. Moreover, from day 15 after vaccination, Group 3 exhibited higher IgG3 and FcγR binding across variants of concerns, including Omicron and its subvariants, compared to the ChAdOx1-boosted individuals. Overall, this highlights the potential of SCB-2019 as a cost-efficient boosting regimen effective across variants of concerns.

## Introduction

SARS-CoV-2 is a highly transmissible and pathogenic coronavirus that emerged in late 2019 and, as of January 30, 2023, has caused 753,001,888 confirmed cases of COVID-19, and 6,807,572 deaths^1^. Remarkable progress has been made in the research and development of COVID-19 vaccines, with the approval and emergency use authorization of numerous vaccines worldwide^2^. Nevertheless, vaccine shortages in low-income countries along with the waning of vaccine-induced immunity across platforms, the emergence of variants of concern (VOCs), and the consequent COVID-19 re-infection cases, underscore the need for newly approved cost-effective vaccines, including protein vaccines.

SARS-CoV-2 protein subunit vaccine candidates, including SCB-2019, have been shown to be safe, well tolerated, highly immunogenic, able to generate persistent immunity and protection, and capable of reducing SARS-CoV-2 transmission. Safety profiles for the SCB-2019 vaccine are competitive with other immunizations^3–7^. SCB-2019 vaccine administered as a homologous or heterologous booster had an acceptable reactogenicity and safety profile with no major safety concerns in the adult study population. The safety profile was comparable to that observed following primary vaccination. Also, SCB-2019 vaccination results in few and quickly resolving adverse events, even at high doses^3,5^. Recent studies have shown that a protein subunit vaccine induces binding and functional humoral responses to several emerging VOCs^8–10^, indicating that protein-based vaccines can also protect against infection from emerging SARS-CoV-2 variants, especially due to the collaboration between antibody Fab and Fc functions^9,10^.

Strong evidence has been generated showing that neutralizing antibodies are mechanistic correlates of protection against SARS-CoV-2 infection^11^. Conversely, disease attenuation is likely dependent on the ability of functional antibodies to operate the clearance of the pathogen. Since effector function-modulating antibodies do not necessarily rely on direct interference with virulence factors, they can bind across the entire Spike antigen surface and opsonize free virus^12–14^. Therefore, while neutralization can be more susceptible to variation in specific virulence factors^15^, non-neutralizing functional antibodies can remain more robust across new emerging variants^16–19^.

Here, we describe the humoral profile including Fc receptor-binding profile, generated by boosting with various formulations of SCB-2019 in ChAdOx1-primed individuals, to assess the potential ability of SCB-2019 in generating non-neutralizing functional antibodies. Compared to ChAdOx1-boosted individuals, SCB-2019-boosted individuals were able to generate a higher FcR binding profile driven by higher IgG3 with a dose-dependent manner. Our study highlights the potential of SCB-2019 boosting in maintaining protection against severe diseases and emerging variants.

## Results

A total of 80 participants primed with 2 doses of the ChAdOx1 vaccine were equally divided into 4 groups with following booster formulations: Group 1: 9 μg SCB-2019 and Alhydrogel; Group 2: 9 μg SCB-2019, CpG 1018, and Alhydrogel; Group 3: 30 μg SCB-2019, CpG 1018, and Alhydrogel; Group 4: ChAdOx1. Blood samples were collected at baseline (day 1), ~15 days after vaccination (day 15), and ~29 days after vaccination (day 29) (see “Methods”). Nine participants (3, 0, 5, and 1 cases in groups 1–4, respectively) were diagnosed with COVID-19 during the first month of the study and removed from the analyses.

There were no statistically significant differences in demographic distribution across groups, except for race and ethnicity. A few more participants were female (56%, 42/75), of the white race (81%, 61/75), and of the Hispanic/Latino ethnicity (73%, 55/75). Mean age was 42.1 years and mean body mass index was 29 kg/m^2^. Details on the demographic distribution of evaluable participants are presented in Table 1.

**Table 1:** List of demographic data of the participants of this study.

		Group				
		G1	G2	G3	G4	All
Sex^a^						
Female	*N*	8	10	15	11	44
	%	44	53	71	61	58
Male	*N*	10	9	6	7	32
	%	56	47	29	39	42
Age (years)^b, c^	Mean	45.4	43.8	41.3	38.6	42.3
	SD	13.5	14.2	13.1	14.9	13.9
Race^d^						
American Indian/Alaska native	*N*	0	0	0	1	1
	%	0	0	0	6	1
Black or African American	*N*	1	2	1	2	6
	%	6	11	5	11	8
Other	*N*	1	1	3	1	6
	%	6	5	14	6	8
Unknown/not reported	*N*	2	0	1	0	3
	%	11	0	5	0	4
White	*N*	14	16	16	14	60
	%	78	84	76	78	79
Ethnic Group^d^						
Hispanic or Latino	*N*	13	16	13	13	55
	%	72	84	62	72	72
Non-hispanic or Latino	*N*	1	1	6	4	12
	%	6	5	29	22	16
Unknown/not reported	*N*	4	2	2	1	9
	%	22	11	10	6	12
Mean body mass index (kg/m^2^)^b,c^	Mean	30.2	28.7	28.5	27.4	28.7
	SD	4.5	5.6	6.0	6.3	5.6
All	*N*	18	19	21	18	76

*SD* standard deviation.

^a^Chi-square test, *P* > 0.05.

^b^Shapiro–Wilk normality test, *P* > 0.05.

^c^Kruskal–Wallis test, *P* > 0.05.

^d^Chi-Square test, *P* < 0.05.

### Systems serology analysis of vaccine arms reveals differences in humoral responses

**Fig. 1:**
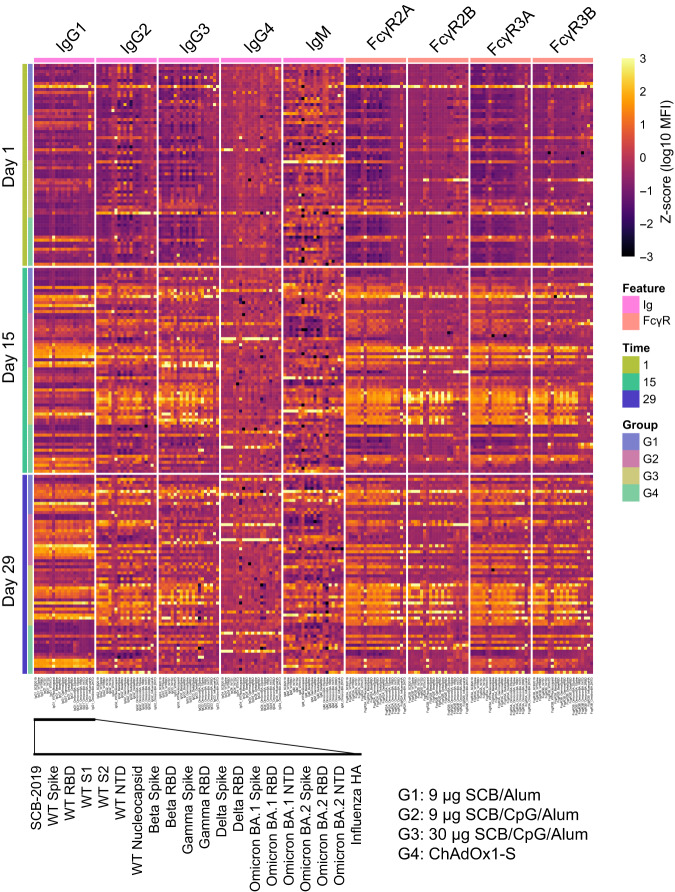
Heatmap summary of antibody binding data by isotype, subclass, and Fcγ receptor-binding. Shown are the binding profiles at baseline, day 15, and day 29 of the various treatment groups. Each row represents the Z-scored binding profile of an individual sample, and each column represents a single antigen (see the zoomed-in view at the bottom). Each isotype, subclass, and FcγR is shown in the same order. Z-scores are calculated across all samples for a single antibody feature. Groups 1, 2, 3, and 4 refer to treatment arms of 9 µg of SCB-2019 + Alum, 9 µg of SCB-2019 + Alum + CpG, 30 µg of SCB-2019 + Alum + CpG, and ChAdOx1, respectively (see “Methods”).

To identify humoral responses to vaccination arms over time, we used systems serology to deeply profile antibody binding patterns. Antibody profiles of IgG1, IgG2, IgG3, IgG4, IgM, and Fc-gamma receptor IIA (FcγRIIA/FcγR2A), FcγRIIB/FcγR2B, FcγRIIIA/FcγR3A, and FcγRIIIB/FcγR3B binding against SARS-CoV-2 antigens were measured (Fig. 1). SARS-CoV-2 antigens include spike and the receptor-binding domain (RBD) of wild-type (WT) and variants of concern (VOCs), including Gamma, Omicron and subvariants including recombinant XBB (Supplementary Table 1). As expected, antibody level and their Fc receptor binding increased with time with the lowest values for isotypes, subclasses, and FcγRs being at baseline, and the highest values being on day 29.

**Fig. 2:**
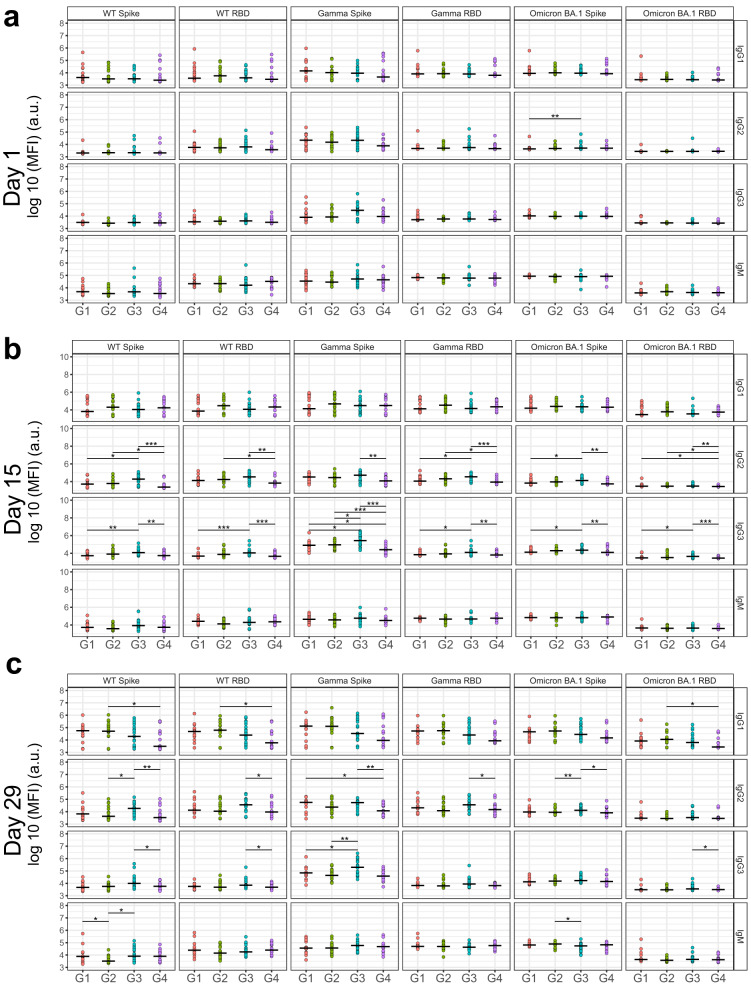
Univariate differences in antibody binding titers among treatment groups. **a** Baseline antibody features against the indicated antigen of Groups 1, 2, 3, and 4 (designated G1, G2, G3, and G4, respectively) are shown against VOC Spike and RBD, as well as WT Spike and RBD. Shown on the *y* axis is the binding median fluorescence intensity (MFI) in log base 10 of the indicated antibody isotype or subclass. **b** Same as (**a**), but for antibody binding profiles at day 15. **c** Same as (**a**), but for antibody binding profiles at day 29. *P < 0.05, **P < 0.01 ***P < 0.001 before multiple test correction (Wilcoxon rank-sum test). Groups 1, 2, 3, and 4 refer to treatment arms of 9 µg of SCB-2019 + Alum, 9 µg of SCB-2019 + Alum + CpG, 30 µg of SCB-2019 + Alum + CpG, and ChAdOx1, respectively. a.u. arbitraray unit.

We performed univariate comparisons of full-length Spike- and RBD antibody binding for IgG1, IgG2, IgG3, and IgM. As expected, titers were minimal across groups on day 1 for all antibody isotypes and subclasses (Fig. 2a and Supplementary Figs. 1a and 3a). However, on day 15, differences in antibody levels were apparent, which were most pronounced for IgG3 in SCB-2019 recipients (Group 3) compared to ChAdOx-1 recipients (Group 4). Specifically, Group 3 displayed a significantly higher IgG3 against Spike and RBD of WT and multiple variants, including Beta, Delta, Gamma, and Omicron BA.1 than Group 4. Importantly, IgG3 responses against other subdomains of Spike were higher in Group 3 than in Group 4, which included the N-terminal domain (NTD) of WT and Omicron BA.1, and the S2 domain of WT (Supplementary Fig. 1), suggesting cross-variant and cross-epitope dominance of IgG3 in Group 3. Notably, IgG2 followed a similar trend with IgG3, but IgG1 did not (Fig. 2b). On day 29, IgG3 continued to trend higher for Group 3 than the Group 4 (Fig. 2c and Supplementary Figs. 1c and 3c). In SCB-2019 groups, dose-dependent increase in responses were observed for IgG3 targeting Spike and RBD of WT and some VOCs (Group 1 vs Group 3 on day 15 and Group 2 vs Group 3 on day 29) (Figs. 2b, c and Supplementary Figs. 1b, c and 3b, c).

While Groups 3 and Group 4 responses were distinct on days 15 and 29, it is unclear if both groups experienced peak response simultaneously. IgG1 titers peaked in Groups 1–3 on day 29, but peaked on day 15 in Group 4. In addition, IgG3 titers peaked in Groups 1–3 on day 15 but on day 29 in Group 4. Still, maximal responses of IgG3 in Group 4 (i.e., responses on day 29) were still lower than the maximal response of IgG3 in Group 3 (i.e., responses on day 15), showing that the observed superiority of IgG3 titers in Group 3 compared to Group 4 are not explained by differential time to reach the peak, at least until day 29 (Supplementary Fig. 5).

### FcγR-binding antibodies display a persistent response in high-dose SCB-2019 arm

**Fig. 3:**
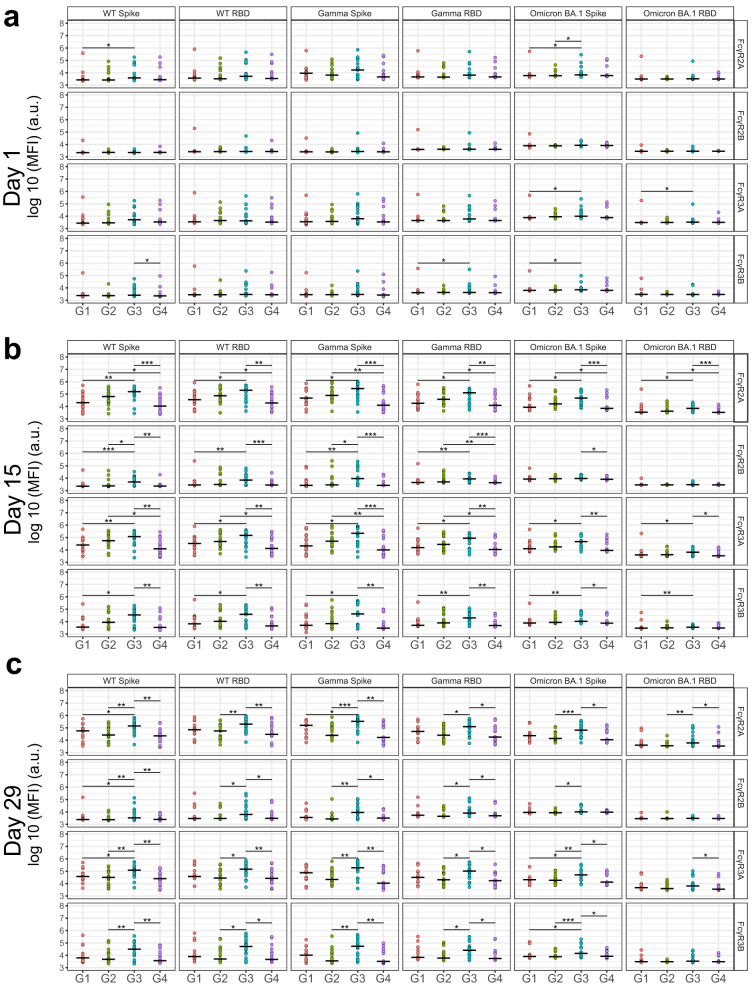
Univariate differences in FcγR-binding antibody titers among treatment groups. **a** Baseline FcγR-binding profiles against the indicated antigens of Groups 1, 2, 3, and 4 (designated G1, G2, G3, and G4, respectively) are shown against VOC Spike and RBD, as well as WT Spike and RBD. Shown on the *y* axis is the binding median fluorescence intensity (MFI) in log base 10 of the indicated FcγR. **b** Same as (**a**), but for FcγR-binding profiles at day 15. **c** Same as (**a**), but for FcγR-binding profiles at day 29. *P < 0.05, **P < 0.01 ***P < 0.001 before multiple test correction (Wilcoxon rank-sum test). Groups 1, 2, 3, and 4 refer to treatment arms of 9 µg of SCB-2019 + Alum, 9 µg of SCB-2019 + Alum + CpG, 30 µg of SCB-2019 + Alum + CpG, and ChAdOx1, respectively. a.u. arbitraray unit.

Non-neutralizing functions of antibodies are mediated through post-translational modifications of the Fc domain, which can bind to low-affinity FcγRs expressed on the surface of immune cells. We thus assayed for antigen-specific FcγR-binding antibodies in our 4 treatment groups which showed little-to-no differences at baseline, as expected (Fig. 3a and Supplementary Figs. 2a and 4a). However, similar to antibody binding titers, FcγR-binding antibodies showed marked differences between Group 3 and Group 4 on day 15. In fact, every FcγR-binding antibody in Group 3 (30 µg of SCB-2019 + CpG + Alum) was significantly higher for WT Spike, WT RBD, Gamma Spike, Gamma RBD, and Omicron BA.1 Spike compared to Group 4 (Fig. 3b). FcγR2A binding response was most robust across variants, with Beta Spike, Beta RBD, Delta Spike, Delta RBD, BA.1 Spike, BA.1 RBD, BA.1 NTD, BA.2 Spike, BA.2 RBD, and BA.2 NTD showing additional significant difference between Group 3 and Group 4. On day 29, higher FcγR-binding antibodies in Group 3 compared to Group 4 generally persisted but with less pronounced differences (Fig. 3c and Supplementary Figs. 2c and 4c).

Increasing dosage and addition of CpG increased FcγR binding for the SCB-2019 arms that appeared in Spike and RBD across variants and S2 domain of WT on day 15 (i.e., Group 1 vs. Group 3, Fig. 3b and Supplementary Figs. 2b and 4b). However, the differences between Groups 1 and 2 or Groups 2 and 3 were not significant. Of note, the significant differences between Groups 1 and 3 disappeared by day 29 while Groups 2 and 3 became significantly different for a few measurements. The comparability of Groups 1 and 2 at all time points suggests that the CpG adjuvant did not improve Fc receptor binding significantly. A higher dose of SCB-2019 (Group 3) conferring higher antibody and Fc receptor binding level persists until day 29.

For all univariate comparisons, Influenza HA was used as a non-specific control. HA-binding antibodies were marginally enriched in some comparisons, but these enrichments formed no overall trend. Moreover, baseline values for anti-HA Ig were consistent across groupings, suggesting that it is likely that no groups had higher or lower baseline Ig levels (Supplementary Fig. 1).

### Multivariate clustering models show IgG3 drives separation between SCB-2019-recipients and ChAdOx-1-recipients

Univariate comparisons revealed significant differences in antibody titers and FcγR binding across groups, which was conserved across VOCs. Thus, we next aimed to examine global differences in humoral response by identifying a feature set optimally effective in separating the treatment groups with multivariate analysis. To that end, Principal Component Analysis (PCA) and Least Absolute Shrinkage and Selection Operator (LASSO)-based Partial Least Square Discriminant Analysis (PLS-DA) were employed (see “Methods”).

**Fig. 4:**
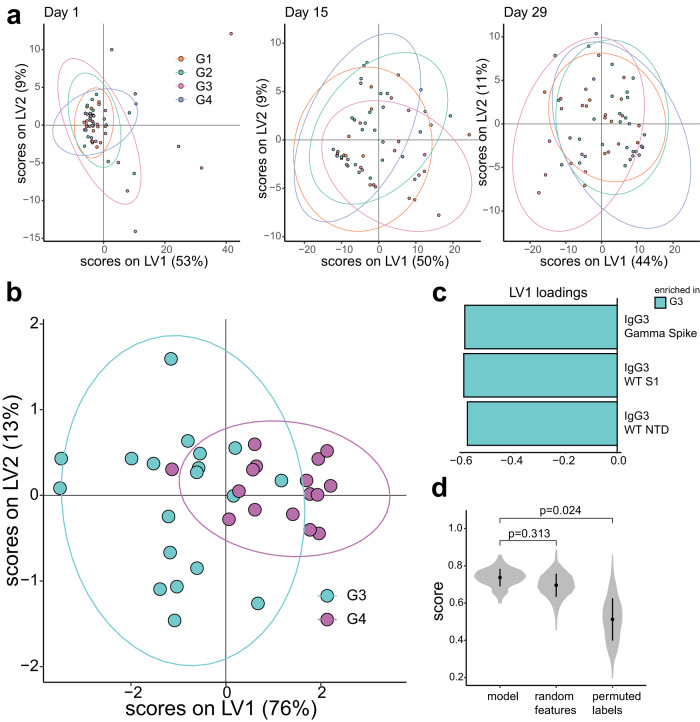
Multivariate clustering of immune profiles. **a** PCA of all data at baseline and 15 and 29 days after vaccination. **b** A supervised PLS-DA model distinguishing G3 vs. G4 at day 15, **c** LV1 loadings for each corresponding PLS-DA. **d** Accuracy distributions over 100 fits of the PLS-DA model with the LASSO-selected features (model), 1000 fits of the PLS-DA model with randomly selected features (random features), and 1000 fits of the PLS-DA model with permuted labels.

**Fig. 5:**
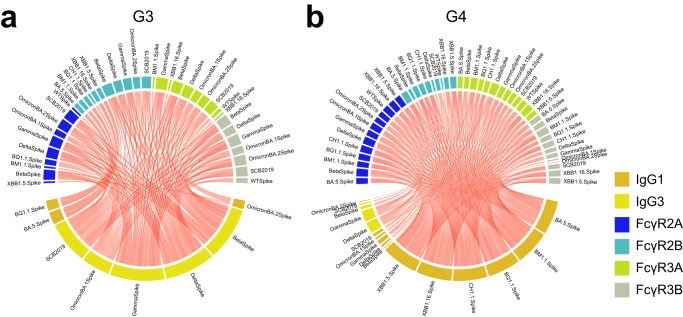
Correlation of selected features on day 15. The chord diagram shows Spearman’s correlations of the features in Group 3 (**a**) and Group 4 (**b**) on day 15. Only spike protein-specific IgG1, IgG3, and FcγR binding were used. Red links indicates correlation between features with Spearman’s rho >0.7 and FDR < 0.05 after multiple test correction (Benjamini–Hochberg procedure) for all comparisons shown in each chord diagram.

Similar to univariate comparisons, multivariate clustering using PCA showed almost complete overlap of the treatment groups at baseline (Fig. 4a). Slight differences appeared on day 15, especially for Group 3 (pink), but the groups showed no noticeable separation. Then, we compared Group 3 (30 µg of SCB-2019 + CpG + Alum) and Group 4 at day 15 using PLS-DA (Figs. 4b, c). IgG3-associated features (enriched in Group 3) were selected by LASSO as representing features that separate between groups, which corroborates findings from univariate comparisons. This model was tested against models built with random features or permutated labels (see “Methods”). The model performed significantly better than permuted label-trained models (Fig. 4d). Yet the model did not significantly outperform models trained on randomly selected features. Similar performance with random feature models suggests that LASSO-selected features do not uniquely describe the separated groups; non-uniqueness in the selected feature is plausible given that many observed features are strongly correlated (Fig. 5), or were distinct between Group 3 and 4 (e.g., IgG3 and FcyR, Supplementary Figs. 1–4, Figs. 2 and 3).

### SCB-2019 vaccine recipients exhibit IgG3 and FcγR-binding responses to highly diverged SARS-CoV-2 Spike variants

We next asked if vaccination with SCB-2019 could induce antibodies capable of recognizing highly diverged SARS-CoV-2 Spikes. These included Omicron-sublineages BA.5, BM.1.1, BQ.1.1, CH.1.1, and recombinant variants XBB.1.5 and XBB.1.16. Magnitudes of responses relative to day 1 to these Spikes were lower for all groups compared to WT and other variants, as expected. However, similar trends were still observed for persistent responses for Group 3 (30 µg of SCB-2019 + CpG + Alum) relative to the other groups. This was most notable for IgG3 toward BQ.1.1 Spike at day 15 (Supplementary Fig. 6) and for FcγR-binding antibodies to BA.5, BM.1.1, BQ1.1, CH1.1, XBB.1.16, and XBB1.5 at day 15 (Supplementary Fig. 7).

Overall, Group 3 yielded the most broadly reactive responses to SARS-CoV-2 Spikes of distal Omicron-sublineages. These responses were elevated for both direct antigen binding and antigen-specific FcγR binding up to 1 month post-vaccination.

### SCB-2019-recipients and ChAdOx1-recipients exhibit IgG3-driven FcγR binding and cross-variants immunity

Given superior IgG3 and FcγR-binding response for multiple variants in Group 3 compared to Group 4, we next asked if IgG3 drives FcγR binding and if this occurs with cross-variants manner. To this end, we calculated correlations between IgG1, IgG3 and FcγR binding for Spikes of all VOCs and sublineages tested (Fig. 5). We focused on day 15 at which we observed the most differences between Group 3 and Group 4 in both univariate and multivariate comparisons. In both Group 3 and Group 4, IgG1 and IgG3 response were correlated with FcγR-binding profile, but Group 4 exhibited more correlations between IgG1 and FcγR binding and Group 3 exhibited more correlations between IgG3 and FcγR binding, corroborating IgG3-focused response in Group 3. Overall, Group 3 and Group 4 had related humoral architectures with both showing IgG1- and IgG3-driven FcγR binding across variants. This suggests that superior IgG3 responses is the main driver for enhancement in FcγR-binding antibodies in Group 3.

## Discussion

The effectiveness of COVID-19 vaccines, as judged by neutralizing antibodies, has decreased as new VOCs have emerged. The Omicron lineage emerged in late 2021 and displayed the greatest degree of neutralizing antibody escape. However, a corresponding drop in protection against severe disease was not observed in vaccinated individuals as Omicron swept throughout the world. This pointed to the non-neutralizing functions of antibodies, as well as cellular immune responses, as key contributors to protection.

Non-neutralizing functions are leveraged through post-translational modifications of the Fc domain of antibodies. These modifications allow for binding and signaling through FcγRs (in the case of IgG subclasses) expressed on the surface of neutrophils, myeloid-lineage cells, natural killer cells, etc. The majority of non-neutralizing activities of IgG subclasses are through modifications of IgG1 and IgG3. Previous reports have demonstrated that non-neutralizing functions were linked to positive clinical outcomes^17,19–21^.

Here we show that IgG, particularly IgG3, was strongly stimulated upon high-dose SCB-2019 immunization. Moreover, FcγR-binding IgGs was strongly stimulated by SCB-2019, and persisted in serum for at least 1 month after immunization. In comparison, IgG titers showed decreased levels even after 1 month following mild infection with SARS-CoV-2^22^. The FcyR-engaging antibodies from our study were able to bind to all VOC Spikes, including Omicron BA.1, BA.2, BA.5, and other Omicron-sublineages and even recombinant lineages such as XBB.1.5 and XBB.1.16. Notably, antibodies generated through SCB-2019 vaccination displayed multi-subdomain binding, demonstrating that this vaccine method can generate antibodies that recognize all major subdomains of Spike. We noted that higher doses of the SCB-2019 yielded the strongest and most durable responses, consistent with previous reports. Importantly, all dosing strategies of this formulation exhibited low adverse event profiles^5^. Therefore, the broadly reactive and functionally leveraged antibodies elicited by the higher dose argue for further investigation into longitudinal responses.

SCB-2019 and ChAdOx1 vaccines differ in several aspects: (1) Spike sequence; (2) Spike concentration; (3) the form in which Spike is presented to the host; (4) inflammatory stimuli; and (5) half-life. In SCB-2019, Spike is produced in a recombinant setting, with sequence modifications that guarantee that the final protein has the three Spike domains in the same conformation as the original viral protein^23^. ChAdOx1, on the other hand, uses an adenoviral system to transfect the transcript for Spike into the host cell, which produces the Spike protein on its surface. As with SCB-2019, the protein structure and subunits coded by ChAdOx1 reproduce those of SARS-CoV-2^24^. Although the protein structures are very similar between the two vaccines, Spike concentrations changed between SCB-2019 groups, and likely also between SCB-2019 and ChAdOx1, which may partially explain differences in outcomes. Groups 1 and 2 corresponded to 9 µg and Group 3, 30 µg Spike. Spike concentrations are hard to evaluate for ChAdOx1 since they depend on the uptake and transcriptional efficiency of the host cells. Likewise, Spike expressed through ChAdOx1 is expressed on the membrane surface of the cells, whereas SCB-2019 Spike is soluble. These different presentations probably lead to differences in MHC I and II presentation, and in T and B cell stimulation. Furthermore, distinctions in post-translational modifications of the Fc might be attributed to the inflammatory agents associated with the vaccines: Alhydrogel, CpG 1018 and innate response to adenovirus. Each of these agents elicits a specific inflammatory environment that may lead to changes in B cell and helper T cell activation, defining IgG subclass and post-translational modifications. While all SCB-2019 groups were associated with the same concentration of Alhydrogel, they differed in that groups 2 and 3 also had CpG 1018 as adjuvant, and that group 3 had an approximately 3 times higher antigen content than groups 1 and 2. Since Fc binding increased in general from groups 1 to 3, CpG and higher SCB-2019 protein doses are likely key in interpreting the SCB-2019 results of the Fc assay. Lastly, antigen half-life may also be associated with differences in immune response. Alhydrogel, for instance, binds to antigens and may protect them from degradation, increasing their half-life. The adenovirus vector does not replicate, so protein expression is finite, but Spike proteins probably have a long half-life in mammalian cells. However, we do not know how half-lives compare between these two platforms.

The findings with the Fc assay are similar to those found by ELISA and the neutralization assay, as presented in Costa Clemens, 2022^6^. Antibody titers by ELISA and the neutralization assay were low at baseline and did not differ between groups, and were similarly high on days 15 and 29, as in our data. Likewise, titers levels were broadly Group 4 ≤ Group 1 < Group 2 < Group 3. ELISA for wild-type Spike and RBD showed significantly higher titers for Group 2 and, especially, Group 3, compared to Group 4. The differences were more pronounced on day 15 but were also seen on day 29. Regarding the neutralization assay, significant differences were found on day 15 between Group 2 or Group 3 and Group 4 using WT, Beta, Delta, Gamma, and Omicron VOCs. Group 3 also differed from Group 4 for most VOCs on day 29. Whereas these profiles are similar to those seen with our data, the latter enabled IgG2 and IgG3 to be identified as main isotypes, FcγR binding to be characterized, as well as specific antibody binding to multiple antigen subtypes. Regardless, it appears that ELISA, neutralization assay, and our Fc assay data (particularly IgG2, IgG3, and FcγR binding) are similar in indicating that: (a) increasing doses of SCB-2019 increase antibody binding (differences between Group 2 and Group 3); (b) addition of CpG 1018 marginally increases antibody binding (differences between Group 1 and Group 2—not significant in most cases); and (c) SCB-2019 plus CpG 1018 leads to higher antibody binding than ChAdOx1.

There are several limitations to this study. We did not have a SCB-2019 group with 30 µg Spike and Alhydrogel alone to control whether dose or adjuvant were the main drivers in the immune response. Moreover, how the molar ratio of antigen:adjuvant functions to shape humoral responses to Spike could not be fully addressed in this study. To that end, future work on adjuvant concentrations and profiling of immune responses at the systems level is desperately needed. In addition, samples were only collected up to day 29. Given the finding of persistent responses at day 29, analyses of later samples and evaluation of Fc receptor binding in antibodies from memory B cells would have been ideal. As mentioned above, differences in an antibody’s Fc receptor binding are due to post-translational modifications, such as antibody Fc glycosylation, which could have been assessed by mass spectrometry. We were not able to discreetly link Fc-modifications to clinical protection due to enrollment sizes and limited sample volumes. As discussed, it is likely that these antibody modifications help prevent severe cases of COVID-19 in vaccinees. On the other hand, severe COVID-19 has also been shown to be limited by T cell response^25^, which we could not evaluate in this trial. Further studies should be designed to follow up on the vaccinated populations to evaluate whether SCB-2019 vaccine recipients are less susceptible to severe COVID-19 and correlate the findings to both Fc subtype/Fc receptor binding and cellular immunity, particularly during Omicron waves. It would also be interesting to understand how and if the Fc response can further be improved by optimizing protein and adjuvant dose and proportion. To focus analysis on global trends, data were not normalized for spike protein dose (molecules per group), or patient-specific total Ig. Bead assays were not normalized for secondary antibody affinity; secondary antibody dilutions were previously optimized, making these distinctions negligible. Statistical analyses were performed to address remaining normalization concerns. Specifically, the comparison is done between the groups which are assumed to be equivalently affected by the absence of normalization. Still, normalization may help understand vaccine response in a nuanced and patient-specific manner.

As SARS-CoV-2 continues to spread throughout the human population, future variants will sweep through regions. This has been exemplified by the emergence of the original Omicron VOC, and later on, subsequent sublineages of this branch. Thus, it is imperative that vaccine platforms provide a broad and functional immune response that can leverage Fc-effector functions. Moreover, a durable response is desired as COVID-19 becomes increasingly endemic. We observed that enhanced Fc receptor-binding responses driven by IgG3, to highly divergent Spikes were stronger and persistent in recipients boosted by 30 μg dose of SCB-2019 + CpG + alum. We conclude that boosting with a high-dose SCB-2019 can elicit a pan-VOC humoral response that provides durable, functionally-primed antibodies.

## Methods

### Trial design

This study involves a subset of participants from a main trial. The main phase 2 trial was observer-blinded, randomized, and controlled with 120 participants, the first 80 of which had samples collected for this study. Participants were assigned to one of four groups (20 participants per group for this study), according to the vaccine received: Group G1: Clover Adjuvanted Recombinant SARS-CoV-2 Trimeric S-protein Subunit Vaccine (SCB-2019, Zhejiang Clover Biopharmaceuticals, Huzhou, China) (9 μg) adjuvanted with 0.75 mg Alhydrogel (Croda Health Care, Thousand Oaks Biopharma, Nantong, China); Group G2: SCB-2019 (9 μg) adjuvanted with 1.5 mg CpG 1018 (Dynavax Technologies, Emeryville, CA, USA) and 0.75 mg Alhydrogel; Group G3: SCB-2019 (30 μg) adjuvanted with 1.5 mg CpG 1018 and 0.75 mg Alhydrogel; and Group G4: ≤ 2.5 × 10^8^ infectious units Chimpanzee Adenovirus encoding the SARS-CoV-2 Spike glycoprotein vaccine ChAdOx1 (Fiocruz, Rio de Janeiro, Brazil). Further details are stated in ref. ^6^.

The sample size was not driven by formal statistical hypothesis testing and was considered sufficient for an accurate descriptive summary of antibody subclass and isotype, and Fc receptor-binding subtype distribution.

Enzyme-linked immunosorbent assay (ELISA) and Virus-neutralizing activity (VNA) results have been previously published^6^. Overall, ELISA and VNA titers increased from G1-G3 and G3 titers were higher than those for G4, indicating that increasing doses of SCB-2019 and the addition of CpG 1018 boost antibody production and neutralization properties. This study corresponded to an exploratory immunogenicity objective: to describe antibody isotype, subclass, and Fc receptor-dependent antibody functions using the Luminex Fc assay.

All personnel involved in the analyses were blinded, as well as the safety monitoring team and the participants. The investigational product syringe was opacified to avoid unblinding the participants. Allocation to study groups was by 1:1:1:1 randomization, block size of eight, using the SAS program as run by the sponsor’s statistician. Three blood draws were done, on days 1, 15 (−2/ + 3 days), and 29 (−2/ + 3 days). The study began in November 2021 and is ongoing. Participants are being monitored for 12 months for safety reasons.

The study sponsor was Instituto D’Or de Ensino e Pesquisa (IDOR), Rio de Janeiro, Brazil. Three sites recruited participants: Natal, Rio de Janeiro, and Porto Alegre, all in Brazil. The study protocol was approved by the National Ethical Review Committee CONEP and the sites’ Ethics Review Committees, as well as by the Brazilian regulatory agency ANVISA and the study was conducted according to Good Clinical Practice guidelines. Participants supplied written informed consent at enrollment.

The study population consisted of healthy or medically stable males and females ≥18 years of age who had been primed with two doses of ChAdOx1 Spike (termed ChAdOx1 throughout the remainder of this manuscript) vaccine six months ( ± four weeks) earlier and given written informed consent. The main exclusion criterion was a previous laboratory-confirmed SARS-CoV-2 infection. Blood samples were taken at baseline, days 15 and 29, with a −2/ + 3 day window. Any confirmed SARS-CoV-2 infection during the study was recorded and samples were censored depending on the day of infection.

The study was registered on ClinicalTrials.gov as NCT 05087368.

### Antibody isotype and Fc receptor-binding profiling

Antibodies from serum samples were analyzed, quantitated, and qualified through a previously established Luminex assay. Briefly, 100 µg of purified antigens (Supplementary Table 1) were coupled to specific bead regions of magnetic Luminex beads (Luminex Corp) through carbodiimide-NHS ester-coupling (ThermoFisher). The antigen-coupled beads were washed, blocked, and subsequently incubated with heat-inactivated plasma samples (56 °C for 30 min) at an appropriate sample dilution (1:100–1:1000 for antibody isotyping, and 1:1000–1:2000 for all FcγRs) overnight in covered 384-well plates with continuous shaking (Greiner Bio-One). Beads were washed with 1× Assay Buffer (1× PBS, 0.1% BSA, 0.05% Tween-20) to remove unbound or non-specifically bound antibodies using the magnetic 384-well HydroSpeed Plate Washer (Tecan) for a total of three washes. Secondary antibodies (see Supplementary Table 1) were blocked using 1× Assay Buffer. Blocked antibodies were then added to the washed beads at a concentration of 1:200 in Assay Buffer and incubated for 1 h at room temperature with continuous shaking. Beads were washed with 1× Assay Buffer three times to remove unbound antibodies using the magnetic 384-well Hydrospeed Plate Washer. After the final wash, the antibody:antigen:beads complexes were resuspended in 40 µL QSOL buffer (Sartorius) and analyzed on the iQue Screener PLUS (Intellicyt/Sartorius). Gates for each bead region are predetermined using previously validated standard operating procedures. Median fluorescence intensities for each region are quantified in replicates, and means between the replicates are reported.

For Fcγ-receptor-binding quantification and qualifications, antibody-containing sera were incubated with antigen-coated beads and processed as above. Custom synthesized Fcγ-receptors (FcγR2A, FcγR2B, FcγR3A, and FcγR3B; Duke Protein Production facility) were biotinylated, then bound to pre-blocked PE-Streptavidin in 1X Assay Buffer. The labeled FcγRs were incubated with sera for 1 h at room temperature with continuous shaking. The beads mixture was then washed 3 times with 1X Assay Buffer using the 384-well Hydrospeed Plate Washer. After the final wash, the antibody:antigen:beads mixture was resuspended in 40 µL of QSOL buffer and analyzed on the iQue Screener PLUS (Intellicyt/Sartorius). All flow cytometry files were background corrected and analyzed using Intellicyt ForeCyt (v8.1).

All antigens and FcγRs were equilibrated in 1X PBS using Zeba-Spin desalting and size exclusion chromatography columns (ThermoFisher). Dilution curves for each antibody isotype, subclass, and FcγR were performed for each antigen to establish a linear range of detection and quality control. A PBS control was added to all plates to establish a lower limit of detection of background. See Gating Strategy figure in Supplementary Fig. 8.

### Statistical analysis

Intention to treat was used for all analyses.

Demographic data normality was analyzed by the Shapiro–Wilk normality test. Continuous variables were presented as means ± standard deviations (SDs), and differences between groups were compared using the Kruskal–Wallis test; categorical variables were presented as counts and percentages; differences between groups were compared using the Chi-Square test. Differences were considered significant for *P* < 0.05. SAS Viya 4 was used for demographic data analyses.

Antibody titers and FcγR-binding antibodies were quantified through flow cytometry and reported in median fluorescence intensity (MFI, arbitrary units). Values were log base 10 transformed before statistical analyses. For statistical tests, two-sided Wilcoxon rank-sum tests were performed to determine if statistically significant difference existed between any two comparisons of groups. *P* values before multiple test corrections were reported in figures, but only differences that showed *P* values < 0.05 after multiple test corrections were discussed in the manuscript. Multiple test corrections were done for each assay (row) in each figure panel, using Benjamini–Hochberg procedure.

To classify and distinguish vaccine groups, we first performed feature selection using Least Absolute Shrinkage and Selection Operator (LASSO)^26^. Over 100 iterations, features selected in more than 90 models were retained for further examination. Selected features were used to train a Partial Least Square Discriminant Analysis (PLS-DA) model^27^ distinguishing vaccine groups. Data were centered and scaled before building the PLS-DA model. Alternative models, random feature model and permuted label model, were generated to assess the PLS-DA model. Random feature model was generated by selecting same number of random features instead of LASSO-selected features. Permuted label model was generated by flipping output labels in the data randomly. We performed fivefold cross-validation 10 times for the original PLS-DA model and 100 times with permuted label model and random feature model, respectively. Then, the whole process was repeated 10 times, resulting in total 100 accuracies from the original model, 1000 accuracies from the random feature model, and 1000 accuracies with permuted label model. Exact *P* values comparing distribution of the accuracies between original model and alternative models were calculated to assess the validity of the original model.

For correlation analyses, Spearman correlations were calculated between antibody feature pairs. Multiple test corrections were performed using the Benjamini–Hochberg procedure with FDR < 0.05, within comparisons in each vaccine group.

All statistical analyses of antibody data were done with R (version 4.1).

### Reporting summary

Further information on research design is available in the Nature Research Reporting Summary linked to this article.

### Supplementary information

Supplementary Material

REPORTING SUMMARY

## Data Availability

Raw data for this manuscript has been deposited on GitHub: https://github.com/RagonSystemSerology/NPJV20231025.

## Code availability

All analyses done in this manuscript are open-source or detailed on GitHub at https://github.com/LoosC/systemsseRology. Full code availability can be obtained through the corresponding author.

## References

[1] WHO Coronavirus (COVID-19) Dashboard. https://covid19.who.int/?mapFilter=cases (2023).

[2] Nagy A, Alhatlani B (2021). An overview of current COVID-19 vaccine platforms. Comput. Struct. Biotechnol. J..

[3] Bravo L (2022). Efficacy of the adjuvanted subunit protein COVID-19 vaccine, SCB-2019: a phase 2 and 3 multicentre, double-blind, randomised, placebo-controlled trial. Lancet.

[4] Richmond PC (2021). Persistence of the immune responses and cross-neutralizing activity with variants of concern following 2 doses of adjuvanted SCB-2019 coronavirus disease 2019 vaccine. J. Infect. Dis..

[5] Richmond P (2021). Safety and immunogenicity of S-Trimer (SCB-2019), a protein subunit vaccine candidate for COVID-19 in healthy adults: a phase 1, randomised, double-blind, placebo-controlled trial. Lancet.

[6] Costa Clemens SA (2022). Homologous and heterologous boosting of the Chadox1-S1-S COVID-19 vaccine with the SCB-2019 vaccine candidate: a randomized, controlled, phase 2 study. Open Forum Infect. Dis..

[7] Tadesse BT (2023). Impact of vaccination with the SCB-2019 coronavirus disease 2019 vaccine on transmission of severe acute respiratory syndrome coronavirus 2 infection: a household contact study in the Philippines. Clin. Infect. Dis..

[8] Ambrosino D (2022). Immunogenicity of SCB-2019 coronavirus disease 2019 vaccine compared with 4 approved vaccines. J. Infect. Dis..

[9] Gorman MJ (2021). Fab and Fc contribute to maximal protection against SARS-CoV-2 following NVX-CoV2373 subunit vaccine with Matrix-M vaccination. Cell Rep. Med..

[10] Kaplonek P (2022). mRNA-1273 vaccine-induced antibodies maintain Fc effector functions across SARS-CoV-2 variants of concern. Immunity.

[11] Gilbert PB (2022). A Covid-19 milestone attained—a correlate of protection for vaccines. New Engl. J. Med..

[12] Goldblatt D, Alter G, Crotty S, Plotkin SA (2022). Correlates of protection against SARS-CoV-2 infection and COVID-19 disease. Immunol. Rev..

[13] Bowman KA (2022). Hybrid immunity shifts the Fc-effector quality of SARS-CoV-2 mRNA vaccine-induced immunity. mBio.

[14] Beaudoin-Bussières G (2022). A Fc-enhanced NTD-binding non-neutralizing antibody delays virus spread and synergizes with a nAb to protect mice from lethal SARS-CoV-2 infection. Cell Rep..

[15] Hachmann NP (2022). Neutralization escape by SARS-CoV-2 Omicron subvariants BA.2.12.1, BA.4, and BA.5. New Engl. J. Med..

[16] Tong X (2023). Waning and boosting of antibody Fc-effector functions upon SARS-CoV-2 vaccination. Nat. Commun..

[17] Zohar T (2020). Compromised humoral functional evolution tracks with SARS-CoV-2 mortality. Cell.

[18] Atyeo C (2020). Distinct early serological signatures track with SARS-CoV-2 survival. Immunity.

[19] Dugan HL (2021). Profiling B cell immunodominance after SARS-CoV-2 infection reveals antibody evolution to non-neutralizing viral targets. Immunity.

[20] Bartsch YC (2021). Humoral signatures of protective and pathological SARS-CoV-2 infection in children. Nat. Med..

[21] Zohar T (2022). Upper and lower respiratory tract correlates of protection against respiratory syncytial virus following vaccination of nonhuman primates. Cell Host Microbe.

[22] Ibarrondo FJ (2020). Rapid decay of anti-SARS-CoV-2 antibodies in persons with mild Covid-19. New Engl. J. Med..

[23] Ma J (2021). Cryo-EM structure of S-Trimer, a subunit vaccine candidate for COVID-19. J. Virol..

[24] Watanabe Y (2021). Native-like SARS-CoV-2 spike glycoprotein expressed by ChAdOx1 nCoV-19/AZD1222 vaccine. ACS Cent. Sci..

[25] Sette A, Crotty S (2021). Adaptive immunity to SARS-CoV-2 and COVID-19. Cell.

[26] Zou H, Hastie T (2005). Regularization and variable selection via the elastic net. J. R. Stat. Soc. Ser. B (Stat. Methodol.).

[27] Barker M, Rayens W (2003). Partial least squares for discrimination. Chemometrics.

