# Supplementary material for: Boosting with adjuvanted SCB-2019 elicits superior Fcγ-receptor engagement driven by IgG3 to SARS-CoV-2 spike
Source: NPJ Vaccines. 2024 Jan 5;9:7. doi: 10.1038/s41541-023-00791-y (PMC10770118; doi:10.1038/s41541-023-00791-y)
Supplement: Supplementary file 1 — Supplementary Material [file 41541_2023_791_MOESM1_ESM.pdf]

Supplementary Table 1

REAGENT or RESOURCE	SOURCE	IDENTIFIER
Anti-Human IgG1-PE	Southern Biotech	9054-09
Anti-human IgG2-PE	Southern Biotech	9070-09
Anti-human IgG3-PE	Southern Biotech	9210-09
Anti-human IgG4-PE	Southern Biotech	9200-09
Anti-human IgM-PE	Southern Biotech	9020-09
Anti-human IgA1-PE	Southern Biotech	9130-09
SARS-CoV-2 WT Spike	Sino Biological	40589-V08H4
SARS-CoV-2 WT S1	Sino Biological	40591-V08H
SARS-CoV-2 WT RBD	Sino Biological	40592-V08H
SARS-CoV-2 WT S2	Sino Biological	40590-V08B
SARS-CoV-2 WT NTD	Sino Biological	40591-V49H
SARS-CoV-2 Beta Spike	Sino Biological	40589-V08B7
SARS-CoV-2 Beta RBD	Sino Biological	40592-V08H59
SARS-CoV-2 Gamma Spike	Sino Biological	40589-V08B10
SARS-CoV-2 Gamma RBD	Sino Biological	40592-V08H86
SARS-CoV-2 Delta Spike	Sino Biological	40589-V08B16
SARS-CoV-2 Delta RBD	Sino Biological	40592-V08H115
SARS-CoV-2 Omicron BA.1 Spike	Sino Biological	40589-V08H26
SARS-CoV-2 Omicron BA.1 RBD	Sino Biological	40592-V08H121
SARS-CoV-2 Omicron BA.1 NTD	Sino Biological	40591-V08H42
SARS-CoV-2 Omicron BA.2 Spike	Sino Biological	40589-V08H28
SARS-CoV-2 Omicron BA.2 RBD	Sino Biological	40592-V08H123
SARS-CoV-2 Omicron BA.2 NTD	Sino Biological	40591-V08H44
SARS-CoV-2 Omicron BA.4/5 Spike	Sino Biological	40589-V08H32
SARS-CoV-2 Omicron BM.1.1 Spike	Sino Biological	40589-V08H43
SARS-CoV-2 Omicron BQ.1.1 Spike	Sino Biological	45089-V08H41
SARS-CoV-2 Omicron CH.1.1 Spike	Sino Biological	40589-V08H46
SARS-CoV-2 Omicron Xbb.1.5 Spike	Sino Biological	40589-V08H45
SARS-CoV-2 Omicron XBB.1.16 Spike	Sino Biological	40589-V08H48
Ebola Virus Glycoprotein	IBT Bioservices	0501-015
Influenza HA	Sino Biological	11687-V08H
SCB-2019	IDOR	Custom
PE-Streptavidin	Agilent Technologies	PB32-10
NHS-Sulfo-LC-LC Kit	ThermoFisher	21435
Zebra-Spin Desalting and Chromatography Columns	ThermoFisher	89882
R Studio V 1.4.1103	RStudio, PBC	Open Source
GraphPad Prism	GraphPad Software, LLC	Ragon Site License
FlowJo V. 10.8	FlowJo, LLC	www.flowjo.com/solutions/flowjo/downloads
iQue Forecyt	Sartorius	60028
iQue Screener Plus	Intellicyt/Sartorius	11811
384-well HydroSpeed Plate Washer	Tecan	30190112

MagPlex Microspheres	Luminex MFG	MC12001-01 (Cataloged by region)
Green Fluorescent Neutravidin Microspheres	ThermoFisher	F8776
Red Fluorescent Neutravidin Microspheres	ThermoFisher	F8775

Supplementary Table 1. List of reagents, resources, and equipment used in this study

Supplementary Figure 1

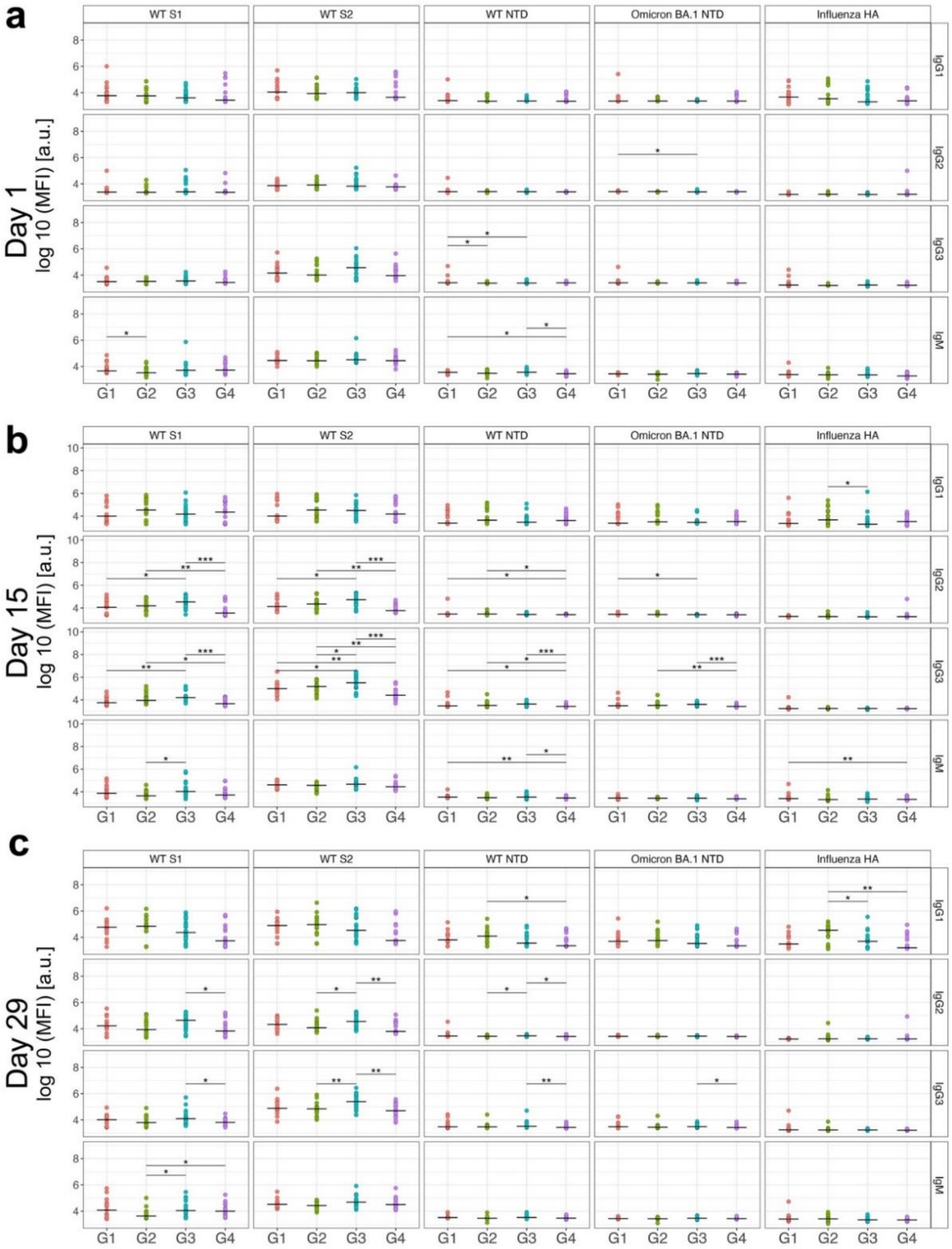

Supplementary Figure 1. Univariate differences in antibody binding titers among treatment groups for Spike subdomains. (a) Baseline antibody binding profiles against the indicated antigen of Groups 1, 2, 3, and 4. Shown on the y-axis is the binding mean fluorescence intensity (MFI) in log base 10 of the indicated antibody isotype or subclass. (b) Same as (a), but for antibody binding profiles at Day 15. (c) Same as (a), but for antibody binding profiles at Day 29. * p<0.05, ** p<0.01 ***p<0.001 before multiple test correction (Wilcoxon rank-sum test). Groups 1, 2, 3, and 4 refer to treatment arms of 9 µg of SCB-2019 + Alum, 9 µg of SCB-2019 + Alum + CpG, 30 µg of SCB-2019 + Alum + CpG, and ChAdOx-1, respectively (see methods). a.u.: arbitrary unit.

Supplementary Figure 2

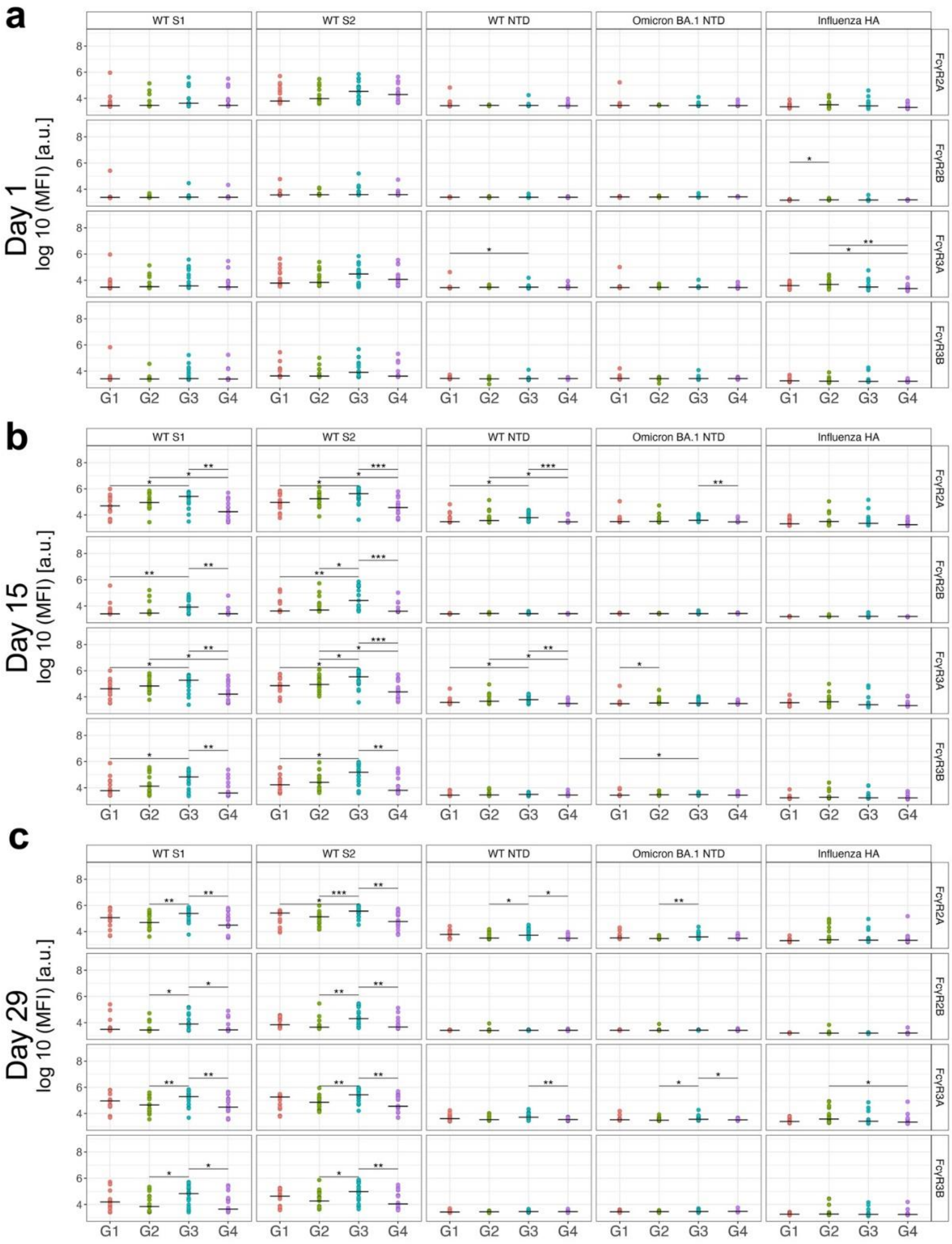

Supplementary Figure 2. Univariate differences in FcγR binding antibody titers among treatment groups for Spike subdomains. (a) Baseline FcγR binding profiles against the indicated antigen of Groups 1, 2, 3, and 4. Shown on the y-axis is the binding mean fluorescence intensity (MFI) in log base 10 of the indicated FcγR. (b) Same as (a), but for FcγR binding profiles at Day 15. (c) Same as (a), but for FcγR binding profiles at Day 29. * $p<0.05$, ** $p<0.01$ *** $p<0.001$ before multiple test correction (Wilcoxon rank-sum test). Groups 1, 2, 3, and 4 refer to treatment arms of 9 μg of SCB-2019 + Alum, 9 μg of SCB-2019 + Alum + CpG, 30 μg of SCB-2019 + Alum + CpG, and ChAdOx-1, respectively (see methods). a.u.: arbitrary unit.

Supplementary Figure 3

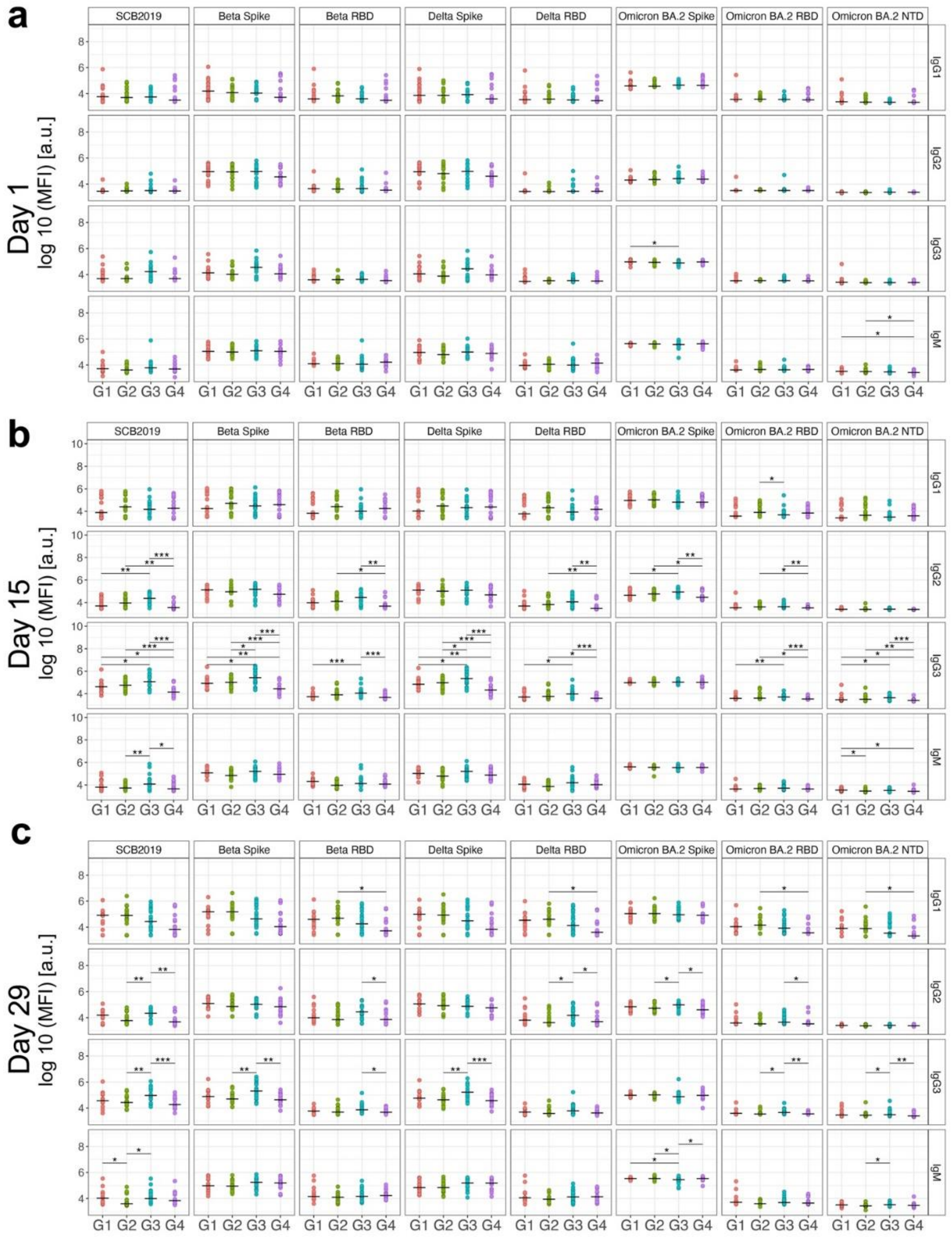

Supplementary Figure 3. Univariate differences in antibody binding titers among treatment groups for VOC Spikes, subdomains, and SCB-2019. (a) Baseline antibody binding profiles against the indicated antigen of Groups 1, 2, 3, and 4. Shown on the y-axis is the binding mean fluorescence intensity (MFI) in log base 10 of the indicated antibody isotype or subclass. (b) Same as (a), but for antibody binding profiles at Day 15. (c) Same as (a), but for antibody binding profiles at Day 29. * $p<0.05$, ** $p<0.01$ ***$p<0.001$ before multiple test correction (Wilcoxon rank-sum test). Groups 1, 2, 3, and 4 refer to treatment arms of 9 µg of SCB-2019 + Alum, 9 µg of SCB-2019 + Alum + CpG, 30 µg of SCB-2019 + Alum + CpG, and ChAdOx-1, respectively (see methods). a.u.: arbitrary unit.

Supplementary Figure 4

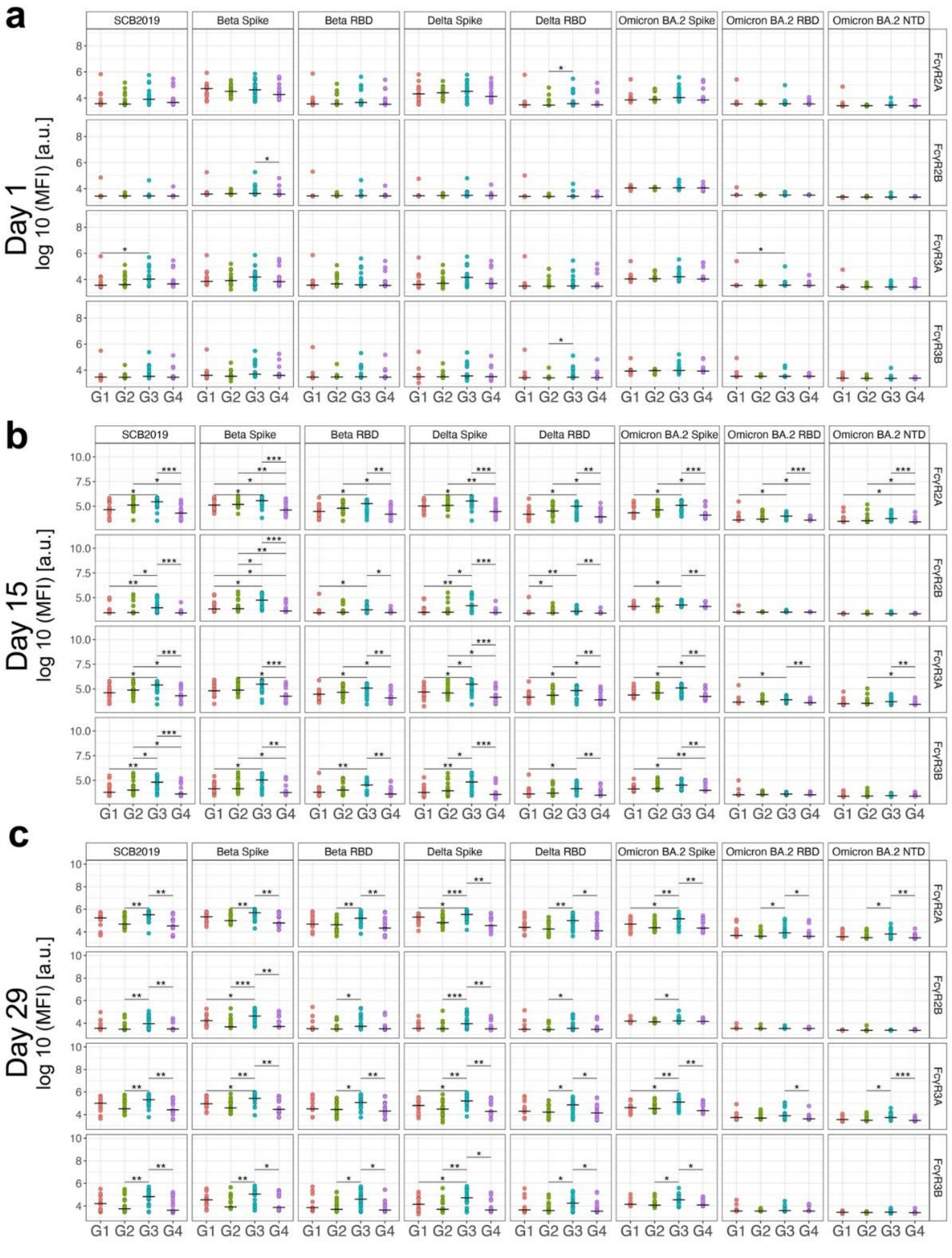

Supplementary Figure 4. Univariate differences in FcγR binding antibody titers among treatment groups for VOC Spikes, subdomains, and SCB-2019. (a) Baseline FcγR binding profiles against the indicated antigen of Groups 1, 2, 3, and 4. Shown on the y-axis is the binding mean fluorescence intensity (MFI) in log base 10 of the indicated FcγR. (b) Same as (a), but for FcγR binding profiles at Day 15. (c) Same as (a), but for FcγR binding profiles at Day 29. * p<0.05, ** p<0.01 ***p<0.001 before multiple test correction (Wilcoxon rank-sum test). Groups 1, 2, 3, and 4 refer to treatment arms of 9 µg of SCB-2019 + Alum, 9 µg of SCB-2019 + Alum + CpG, 30 µg of SCB-2019 + Alum + CpG, and ChAdOx-1, respectively (see methods). a.u.: arbitrary unit.

Supplementary Figure 5

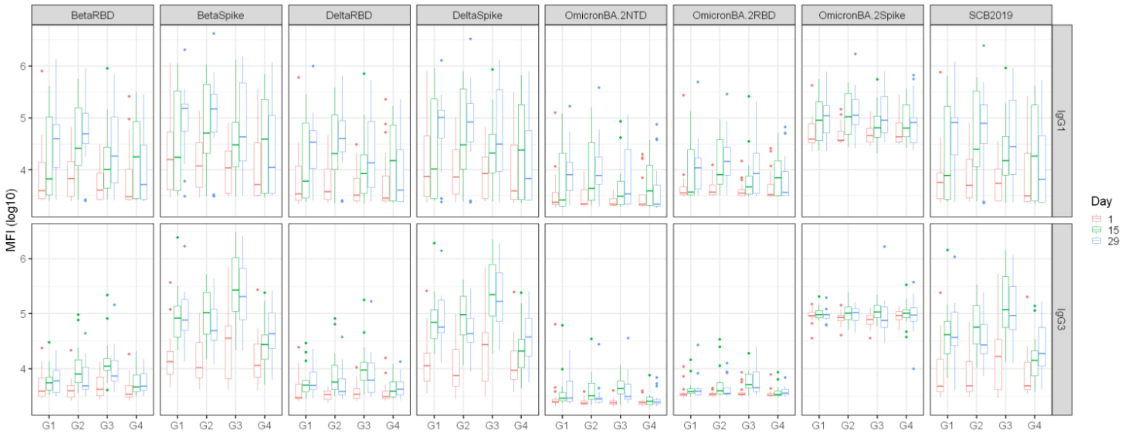

Supplementary Figure 5. Peak response of IgG1 and IgG3 titer across multiple VOCs. Boxplots of log10 mean fluorescence intensity (MFI) measurements for IgG1 and IgG3 antibody specificity across Beta, Delta, Omicron BA.2, and SCB-2019 antigens. Groups 1-4 refer to treatment arms of 9 µg of SCB2019 + Alum, 9 µg of SCB2019 + Alum + CpG, 30 µg of SCB2019 + Alum + CpG, and ChAdOx-1, respectively.

Supplementary Figure 6

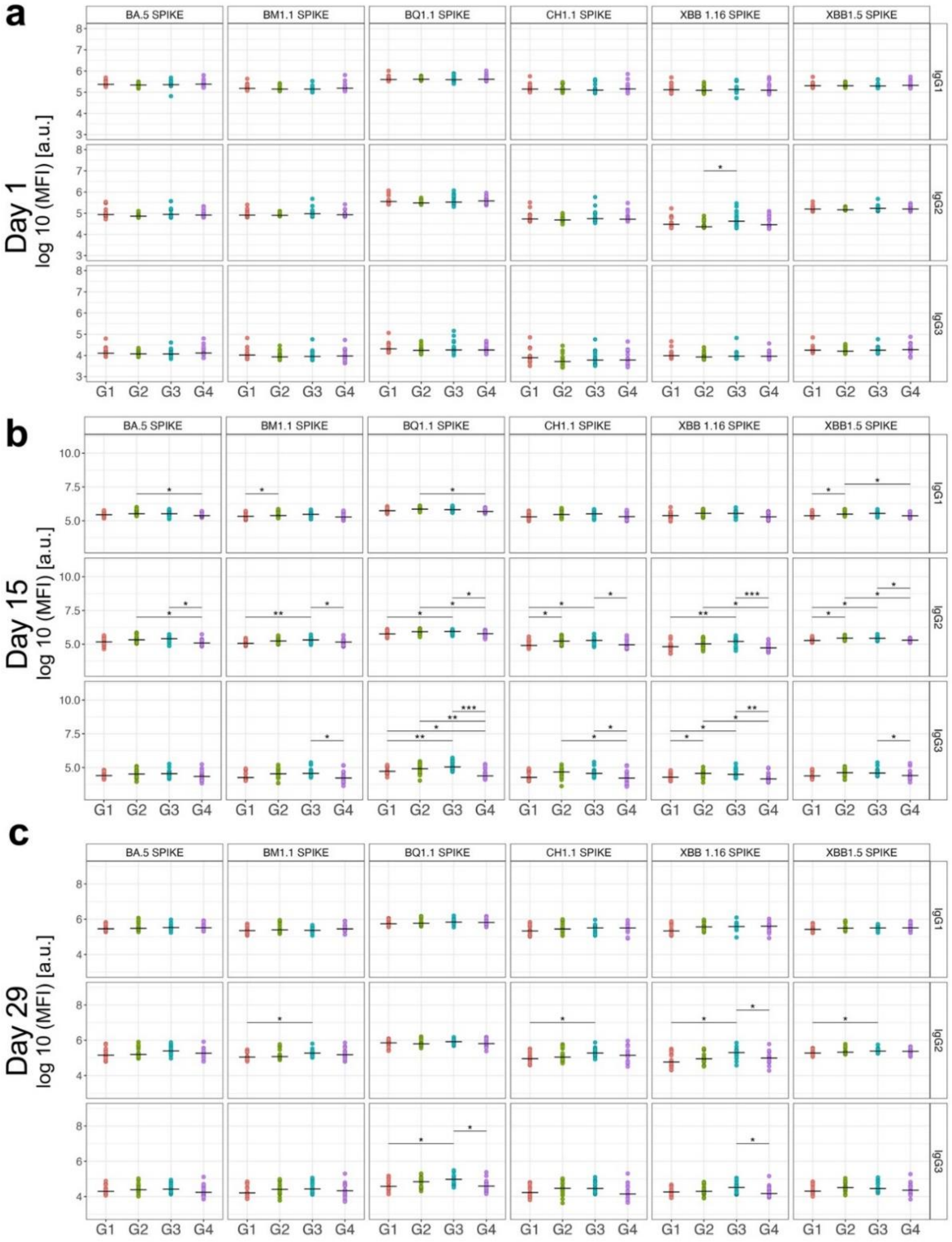

Supplementary Figure 6. Univariate differences in antibody binding titers among treatment groups for Omicron-lineage Spikes. (a) Baseline antibody binding profiles against the indicated antigen of Groups 1, 2, 3, and 4. Shown on the y-axis is the binding mean fluorescence intensity (MFI) in log base 10 of the indicated antibody isotype or subclass. (b) Same as (a), but for antibody binding profiles at Day 15. (c) Same as (a), but for antibody binding profiles at Day 29. * $p<0.05$, ** $p<0.01$ ***$p<0.001$ before multiple test correction (Wilcoxon rank-sum test). Groups 1, 2, 3, and 4 refer to treatment arms of 9 μg of SCB-2019 + Alum, 9 μg of SCB-2019 + Alum + CpG, 30 μg of SCB-2019 + Alum + CpG, and ChAdOx-1, respectively (see methods). a.u.: arbitrary unit.

Supplementary Figure 7

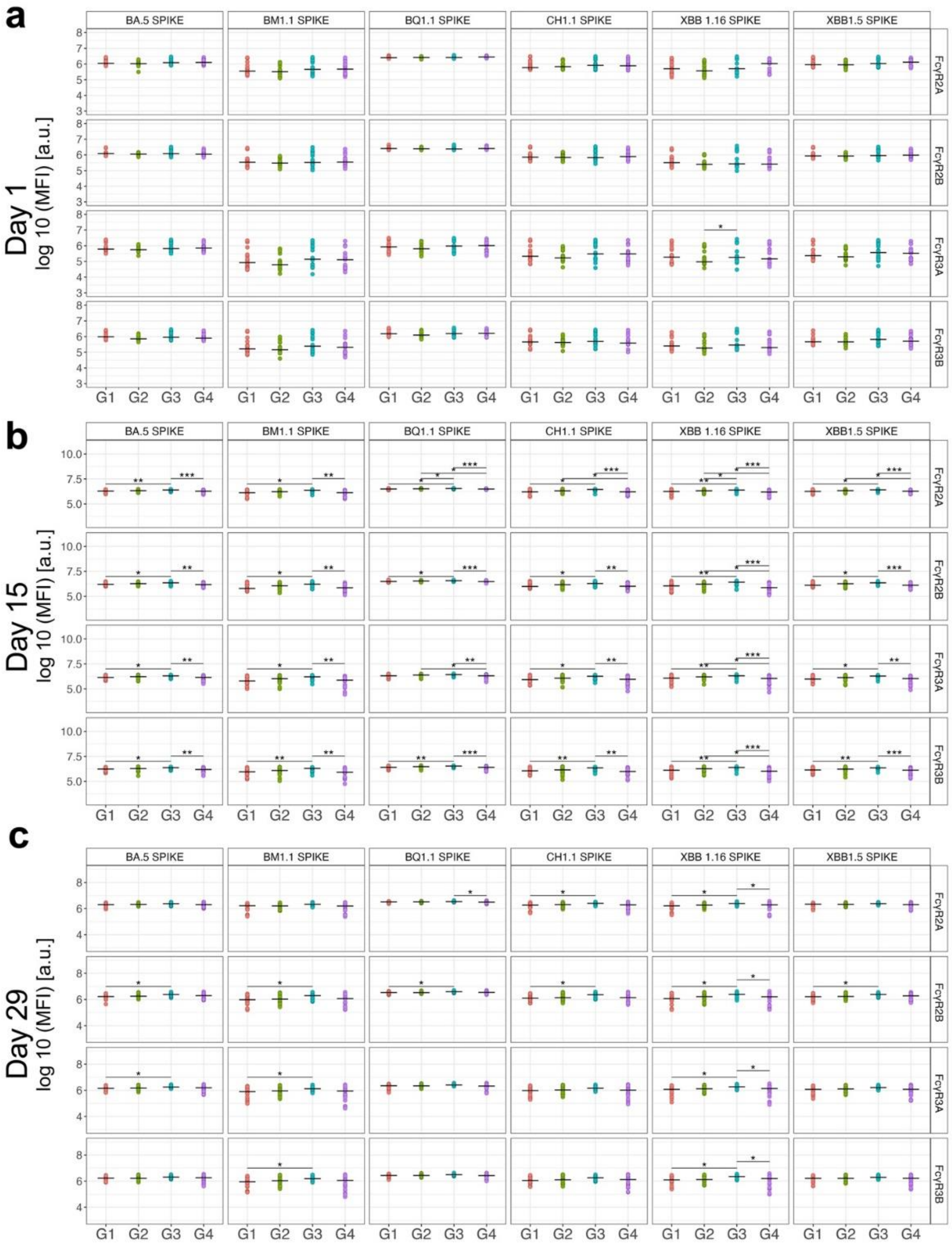

Supplementary Figure 7. Univariate differences in FcγR binding antibody titers among treatment groups for Omicron-lineage Spikes. (a) Baseline FcγR binding profiles against the indicated antigen of Groups 1, 2, 3, and 4. Shown on the y-axis is the binding mean fluorescence intensity (MFI) in log base 10 of the indicated FcγR. (b) Same as (a), but for FcγR binding profiles at Day 15. (c) Same as (a), but for FcγR binding profiles at Day 29. * $p<0.05$, ** $p<0.01$ ***$p<0.001$ before multiple test correction (Wilcoxon rank-sum test). Groups 1, 2, 3, and 4 refer to treatment arms of 9 µg of SCB-2019 + Alum, 9 µg of SCB-2019 + Alum + CpG, 30 µg of SCB-2019 + Alum + CpG, and ChAdOx-1, respectively (see methods). a.u.: arbitrary unit.

Supplementary Figure 8

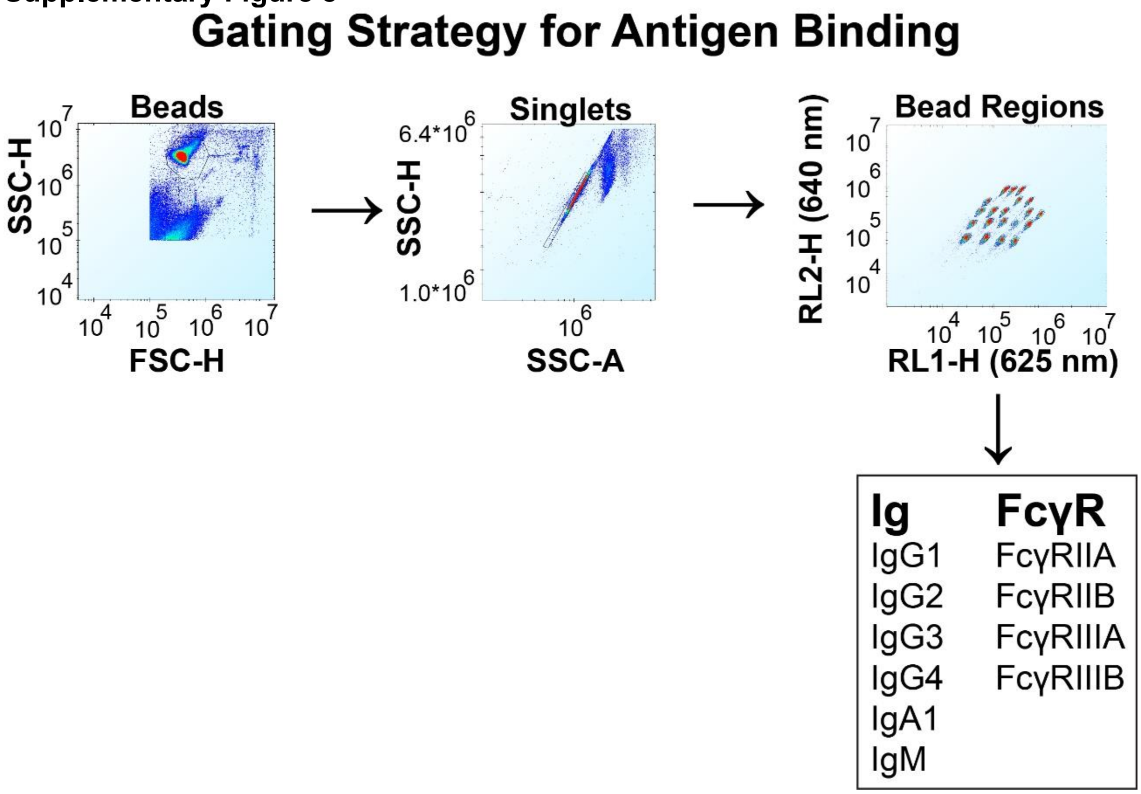

Supplementary Figure 8. Gating strategy for antibody isotype and subclass, and FcγR-binding antibodies. Luminex beads were gated for singlets, and then identified by individual beads regions corresponding to a single antigen. Individual antibody isotypes and subclasses, and FcγR-binding antibodies were quantified for binding to the antigen-coated beads.
