# Supplementary material for: Boosting with adjuvanted SCB-2019 elicits superior Fcγ-receptor engagement driven by IgG3 to SARS-CoV-2 spike
Source: NPJ Vaccines. 2024 Jan 5;9:7. doi: 10.1038/s41541-023-00791-y (PMC10770118; doi:10.1038/s41541-023-00791-y)
Supplement: Supplementary file 2 — REPORTING SUMMARY [file 41541_2023_791_MOESM2_ESM.pdf]

nature portfolio

Corresponding author(s): Ryan P. McNamara

Last updated by author(s): Nov 29, 2023

Reporting Summary

Nature Portfolio wishes to improve the reproducibility of the work that we publish. This form provides structure for consistency and transparency in reporting. For further information on Nature Portfolio policies, see our Editorial Policies and the Editorial Policy Checklist.

Statistics

For all statistical analyses, confirm that the following items are present in the figure legend, table legend, main text, or Methods section.

n/a	Confirmed	
☐	☒	The exact sample size (*n*) for each experimental group/condition, given as a discrete number and unit of measurement
☐	☒	A statement on whether measurements were taken from distinct samples or whether the same sample was measured repeatedly
☐	☒	The statistical test(s) used AND whether they are one- or two-sided<br>*Only common tests should be described solely by name; describe more complex techniques in the Methods section.*
☐	☒	A description of all covariates tested
☐	☒	A description of any assumptions or corrections, such as tests of normality and adjustment for multiple comparisons
☐	☒	A full description of the statistical parameters including central tendency (e.g. means) or other basic estimates (e.g. regression coefficient) AND variation (e.g. standard deviation) or associated estimates of uncertainty (e.g. confidence intervals)
☐	☒	For null hypothesis testing, the test statistic (e.g. *F*, *t*, *r*) with confidence intervals, effect sizes, degrees of freedom and *P* value noted<br>*Give P values as exact values whenever suitable.*
☒	☐	For Bayesian analysis, information on the choice of priors and Markov chain Monte Carlo settings
☒	☐	For hierarchical and complex designs, identification of the appropriate level for tests and full reporting of outcomes
☐	☒	Estimates of effect sizes (e.g. Cohen's *d*, Pearson's *r*), indicating how they were calculated

Our web collection on statistics for biologists contains articles on many of the points above.

Software and code

Policy information about availability of computer code

Data collection	All data in this manuscript was generated on the Intellicyt IQue Screener PLUS with iQue ForeCyt Version 10.0.8341 - build date 11/02/2022
Data analysis	All data analysis in this manuscript was done using R Studio Version 4.1.

For manuscripts utilizing custom algorithms or software that are central to the research but not yet described in published literature, software must be made available to editors and reviewers. We strongly encourage code deposition in a community repository (e.g. GitHub). See the Nature Portfolio guidelines for submitting code & software for further information.

Data

Policy information about availability of data

All manuscripts must include a data availability statement. This statement should provide the following information, where applicable:

- Accession codes, unique identifiers, or web links for publicly available datasets
- A description of any restrictions on data availability
- For clinical datasets or third party data, please ensure that the statement adheres to our policy

Raw data for this manuscript has been deposited on GitHub: https://github.com/RagonSystemSerology/NPJV20231025.

Research involving human participants, their data, or biological material

Policy information about studies with human participants or human data. See also policy information about sex, gender (identity/presentation), and sexual orientation and race, ethnicity and racism.

Reporting on sex and gender	Sex was considered in the recruitment of all cohorts, and further information can be found within the main text as well as at ClinicalTrials.gov under NCT 05087368. For all analyses, staff at the Ragon Institute were blinded to processing of samples, and only after completion of all experiments did unblinding occur. This includes for background information on sex and gender.
Reporting on race, ethnicity, or other socially relevant groupings	Race was self-reported by participants. For all analyses, staff at the Ragon Institute were blinded to processing of samples, and only after completion of all experiments did unblinding occur. This includes for background information such as race, ethnicity, and other social groupings.
Population characteristics	Population characteristics of all groups are summarized in Table 1. This includes a subgroup breakdown of the age ranges, sex, race, and body mass index for all subgroups.
Recruitment	The main phase 2 trial was observer-blinded, randomized, and controlled with 120 participants, the first 80 of which had samples collected for this study. Participants were assigned to one of four groups (20 participants per group for this study), according to the vaccine received: Group G1: Clover Adjuvanted Recombinant SARS-CoV-2 Trimeric S-protein Subunit Vaccine (SCB-2019, Zhejiang Clover Biopharmaceuticals, Huzhou, China) (9 μg) adjuvanted with 0.75 mg Alhydrogel (Croda Health Care, Thousand Oaks Biopharma, Nantong, China); Group G2: SCB-2019 (9 μg) adjuvanted with 1.5 mg CpG 1018 (Dynavax Technologies, Emeryville, CA, USA) and 0.75 mg Alhydrogel; Group G3: SCB-2019 (30 μg) adjuvanted with 1.5 mg CpG 1018 and 0.75 mg Alhydrogel; and Group G4: ≤ 2.5 × 108 infectious units Chimpanzee Adenovirus encoding the SARS-CoV-2 Spike glycoprotein vaccine ChAdOx-1 (Fiocruz, Rio de Janeiro, Brazil). Further information on recruitment can be found at PMID: 36043184.
Ethics oversight	The original study protocol was approved by participating hospital's ethical review committee. Further information can found at PMID: 36043184. Secondary use determination was done by the Mass General Brigham Institutional Review Board (IRB). Processing was done using protocol #2022P001632.

Note that full information on the approval of the study protocol must also be provided in the manuscript.

Field-specific reporting

Please select the one below that is the best fit for your research. If you are not sure, read the appropriate sections before making your selection.

☒ Life sciences ☐ Behavioural & social sciences ☐ Ecological, evolutionary & environmental sciences

For a reference copy of the document with all sections, see nature.com/documents/nr-reporting-summary-flat.pdf

Life sciences study design

All studies must disclose on these points even when the disclosure is negative.

Sample size	The sample size was 120 participants, the first 80 of which had samples collected for this study. Four groups were created, each with 20 participants per group.
Data exclusions	Four individuals tested positive for SARS-CoV-2 during this study, and were removed from further analysis.
Replication	All antibody-binding assays were done in technical replicates using our previously validated assay for SARS-CoV-2 antigen binding (PMID: 37443074, 37000623, 37535402, 36351430, 34824251). Linear ranges of detection were initially established by performing dilutions of serum for each antibody subclass, isotype, and FcR-binding antibody. Upon identification of an appropriate dilution, binding assays were run on a single instrument to avoid instrument-to-instrument variability.
Randomization	Randomization of the initial trial was done per protocol NCT 05087368. Samples were then approved for secondary use by the Mass General Brigham IRB using protocol #2022P001632.
Blinding	Investigators at the Ragon Institute who performed all of the antibody binding profiling were blinded during processing. Only after completion of all experiments and a datasheet was reported to collaborators was the team at Ragon unblinded. This was done to ensure proper and ethical handling of samples.

Reporting for specific materials, systems and methods

We require information from authors about some types of materials, experimental systems and methods used in many studies. Here, indicate whether each material, system or method listed is relevant to your study. If you are not sure if a list item applies to your research, read the appropriate section before selecting a response.

Materials & experimental systems

n/a	Involved in the study
☐	☒ Antibodies
☒	☐ Eukaryotic cell lines
☒	☐ Palaeontology and archaeology
☒	☐ Animals and other organisms
☐	☒ Clinical data
☒	☐ Dual use research of concern
☒	☐ Plants

Methods

n/a	Involved in the study
☒	☐ ChIP-seq
☐	☒ Flow cytometry
☒	☐ MRI-based neuroimaging

Antibodies

Antibodies used	Antibodies used in this study include:<br>Anti-human IgG1-PE from Southern Biotech, Cat# 9054-09<br>Anti-human IgG2-PE from Southern Biotech, Cat # 9070-09<br>Anti-human IgG3-PE from Southern Biotech, Cat #9210-09<br>Anti-human IgG4-PE from Southern Biotech, Cat #9200-09<br>Anti-human IgM-PE from Southern Biotech, Cat #9020-09<br>Anti-human IgA1-PE from Southern Biotech, Cat #9130-09
Validation	Each of these antibodies has been previously validated in the manuscripts PMID: 37443074, 37000623, 37535402, 36351430, 34824251, 35430229, 34652962, 35090580, 35881018).

Clinical data

Policy information about clinical studies
All manuscripts should comply with the ICMJE guidelines for publication of clinical research and a completed CONSORT checklist must be included with all submissions.

Clinical trial registration	Original Clinical Trial identifier NCT05087368
Study protocol	Secondary use protocol #2022P001632.
Data collection	Data collection took place between August 2022 - September 2023 (secondary use period). Original clinical trial was conducted December 2021 - April 2022.
Outcomes	Clinical outcomes were not measured in this study. For the original clinical trial, outcomes were immunogenicity and reactogenicity (see NCT05087368).

Plants

Seed stocks	Not applicable
Novel plant genotypes	Not applicable
Authentication	Not applicable

Flow Cytometry

Plots

Confirm that:

☒ The axis labels state the marker and fluorochrome used (e.g. CD4-FITC).

☒ The axis scales are clearly visible. Include numbers along axes only for bottom left plot of group (a 'group' is an analysis of identical markers).

☒ All plots are contour plots with outliers or pseudocolor plots.

☒ A numerical value for number of cells or percentage (with statistics) is provided.

Methodology

Sample preparation	Plasma samples were approved for secondary use and for exploratory analyses of immune reactivity. Individual antigens were coupled to carbosy luminex beads on an individual region through carbodiimide-NHS ester coupling. Unbound antigen was washed away using 1X PBS.<br>Plasma samples were heat-inactivated at 56C for 30 minutes and centrifuged to remove aggregates. Upper layer was transferred to a 96 well plate and samples were further diluted in 1X PBS. The diluted plasma was incubated with antigen-coated beads in 384-well lo-bind plates with continuous shaking at 4C overnight. Unbound material was washed using 1X assay buffer (1X PBS, 0.1% BSA, 0.05% Tween-20) using the HydroSpeed 384-well plate washer for a total of three washes.<br>Specific antibody isotypes, subclasses, and FcR-binding antibodies were assayed for affinity to the various antigens through the addition of PE-conjugated detection antibodies (Southern Biotech) or PE-conjugated purified human FcRs obtained from Duke University. The beads were incubated with the detection antibody or detection FcR at room temperature for 1 hour with continuous shaking. Unbound material was then washed away using 1X Assay buffer on the HydroSpeed 384-well plate washer for a total of 3 washes<br>After the final wash, 40 uL of QSOL buffer (Intellicyt) was added to each well to resuspend the beads. Plates were then run on the Intellicyt IQue Screener Plus previously described multiplexed flow cytometry workflow (PMID: 23023091). Binding was quantified through IQue ForeCyt and values are reported as the mean fluorescence intensity.
Instrument	All samples were run on the Intellicyt iQue Screener Plus.
Software	Initial flow cytometry files were collected using iQue ForeCyt Version 10.0.8341. Data was exported as .csv files and then analyzed using R Studio V4.1.
Cell population abundance	Gating was set per our previously validated assay originally developed (PMID: 23023091, 28163018, 21192942, 31301278) and subsequently validated for SARS-CoV-2 antigens (PMID: 37443074, 37000623, 37535402, 36351430, 34824251, 35430229, 34652962, 35090580, 35881018).
Gating strategy	Gating strategy was built in a similar manner to PMID: 37443074, but with more antigens. A gating strategy figure has been provided in the Supplemental Information as Supplementary Figure 8.

☒ Tick this box to confirm that a figure exemplifying the gating strategy is provided in the Supplementary Information.
